# Amide-containing neoepitopes: the key factor in the preparation of hapten-specific antibodies and a strategy to overcome

**DOI:** 10.3389/fimmu.2023.1144020

**Published:** 2023-06-05

**Authors:** Xiangning Han, Hong Lin, Xiangfeng Chen, Luefeng Wang, Ziang Zhang, Xiaojing Wei, Xun Sun, Hanyi Xie, Tushar Ramesh Pavase, Limin Cao, Jianxin Sui

**Affiliations:** ^1^ College of Food Science and Engineering, Ocean University of China, Qingdao, China; ^2^ Shandong Analysis and Test Center, Qilu University of Technology (Shandong Academy of Sciences), Jinan, China

**Keywords:** amide-containing neoepitopes, efficiency of antibody preparation, complete antigen, EDC, antibody against small molecules

## Abstract

For a long time, people have suffered from uncertainty, complexity, and a low success rate in generating and screening antibodies against small molecules, which have become the core bottlenecks of immunochemistry. Here, the influence of antigen preparation on antibody generation was investigated at both molecular and submolecular levels. Neoepitopes (amide-containing neoepitopes) formed in the preparation of complete antigens are one of the most important factors limiting the efficiency of generating hapten-specific antibodies, which was verified by different haptens, carrier proteins, and conjugation conditions. Amide-containing neoepitopes present electron-dense structural components on the surface of prepared complete antigens and, therefore, induce the generation of the corresponding antibody with much higher efficiency than target hapten. Crosslinkers should be carefully selected and not overdosed. According to these results, some misconceptions in the conventional anti-hapten antibody production were clarified and corrected. By controlling the content of 1-(3-dimethylaminopropyl)-3-ethylcarbodiimide hydrochloride (EDC) during the synthesis of immunogen to limit the formation of amide-containing neoepitopes, the efficiency of hapten-specific antibody generation could be significantly improved, which verified the correctness of the conclusion and provided an efficient strategy for antibody preparation. The result of the work is of scientific significance in the preparation of high-quality antibodies against small molecules.

## Introduction

Control of small molecules such as pesticides, veterinary drugs, hormones, and persistent environmental pollutants is the spotlight of many fields, including food safety control, biomedical diagnosis, and environmental monitoring (1–5). The challenge lies in the detection of small molecules due to sensitivity limitations. Various immunoassays have been used in the field with excellent sensitivity, simplicity, and specificity (6–8). Successful preparation of high-quality antibodies is the hallmark of the detection of these small molecules (9–11). Unlike proteins or other large molecules, small molecular targets usually lack immunogenicity and must be coupled to carrier proteins to form conjugates, which are then used as immunization antigens to generate antibodies or coating antigens in corresponding immunoassays (12, 13). Until now, 1-(3-dimethylaminopropyl)-3-ethylcarbodiimide hydrochloride (EDC) and *N*-hydroxysuccinimide (NHS)-induced polycondensations have been the most common methods for preparing antigens through the amino-carboxyl covalent bonds between haptens and carrier proteins (14–16). Carriers (usually bovine albumin and ovalbumin) are generally pretreated with ethylenediamine (EDA) or other chemicals to convert amino acid carboxyl groups into amino groups to increase ratios of hapten in conjugates and prevent possible protein self-crosslinking (17, 18). These modified proteins are often referred to as cationized proteins (cBSA or cOVA).

Such a strategy has been employed for decades, but immunization efficiency has exhibited significant uncertainty among properties with different haptens (19–21). In many cases, researchers have been faced with tedious and inefficient screening processes and are even confused by “The titers of antibodies were quite high, but they had low affinity for small molecules” (17, 18, 22). Successful preparation of highly specific antibodies against small molecules is challenging (23–26). These drawbacks have raised increasing questions regarding the rationality and perfection of conventional methods.

In some previous studies, the sensitivity of antiserum has been shown to be improved by antigen affinity purification (27, 28) or by optimizing the length and structure of linkers of immunogen or coating antigens (29–32). A plausible explanation for the observed low immunization efficiency can be proposed: neoepitopes are produced during the conjugation of hapten and carrier, which could induce the generation of large numbers of antibodies recognizing neither hapten nor carrier. Regrettably, there has been no detailed information verifying the aforementioned proposals or providing clarification on the aforementioned phenomenon. Given the increasing demand for preparing specific antibodies against small molecules more quickly and economically, comprehensive clarification of the problems and a subsequent significant improvement of the antibody production strategy seem of great scientific significance.

As pesticides and veterinary drugs can reach food at any stage of processing, their presence has become a growing concern (33). Residues of veterinary drugs such as enrofloxacin (ENR) and norfloxacin (NOF) and pesticide residues such as bifenthrin are widely used in food. Due to its high throughput, high specificity, robustness, and ease of automation, indirect competitive enzyme-linked immunosorbent assay (ic-ELISA) is the most used immunoassay to detect pesticides and veterinary drugs. However, conventional ic-ELISA often suffers from relatively low sensitivity. High-affinity antibodies toward residues are the headstone for sensitivity (6).

In our previous studies, the prepared antisera against many kinds of drug residues showed “high titers but poor half-inhibitory concentration (IC_50_)” are common (**Table 1**). We have had little success in replacing the immunogen with BSA. Therefore, in this study, the influence of protein–hapten conjugation on antibody production was investigated at both molecular and submolecular levels. The importance of the formation of neoepitopes during antigen preparation was revealed and validated for the first time. The mechanism to influence antibody generation was fully discussed, and new strategies for efficiently producing hapten-specific antibodies were explored.

**Table 1:** The information of polyclonal antibodies to five different residues.

Residues	Hapten Structure	Immunogen	Coating antigen	Animal	^a^Titer	^b^IC_50_(ng/mL)
Enrofloxacin (ENR)	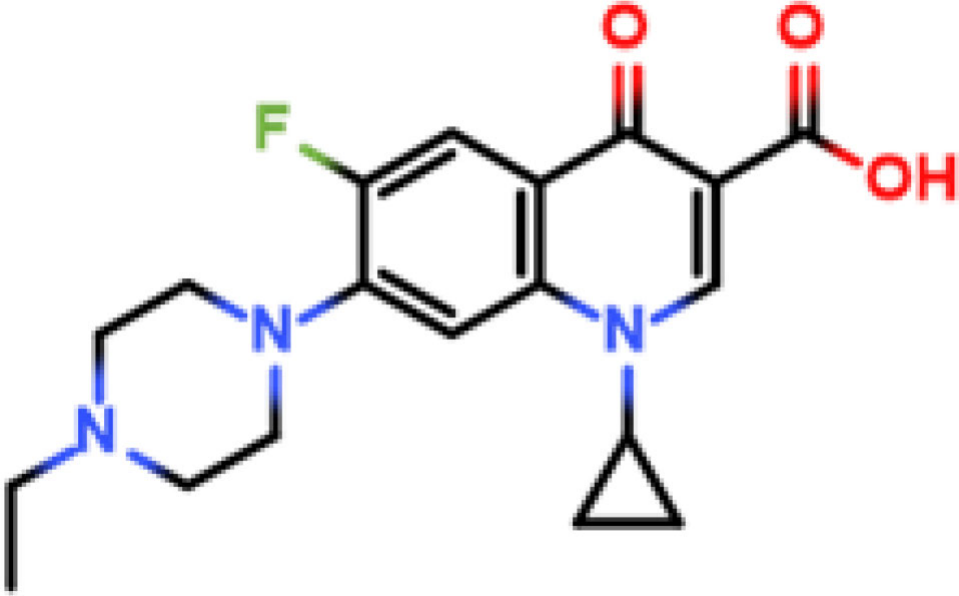	ENR-cOVA	ENR-cBSA	Rabbit	① 1:400000 ② 1:800000 ③ 1:800000	① 766.7 ② >1000 ③ >10000
Norfloxacin (NOF)	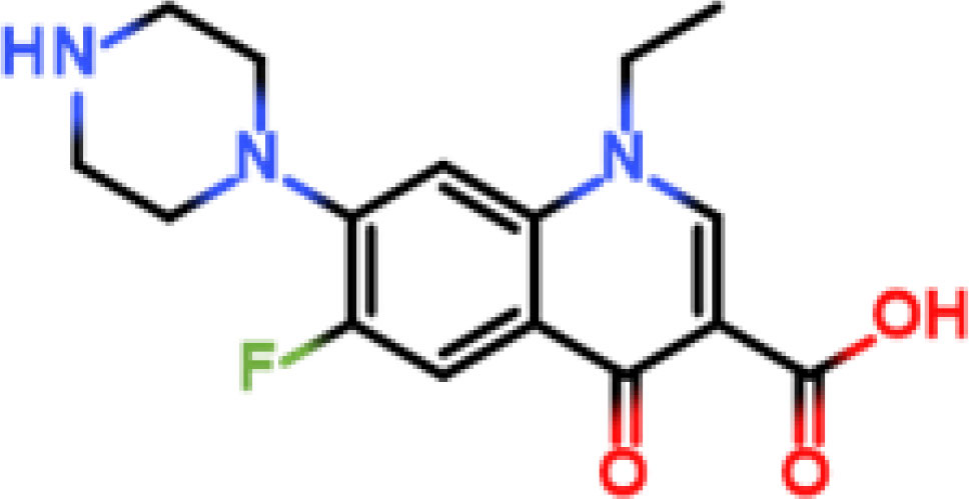	NOF-cOVA	NOF-cBSA	Mouse	① 1:200000 ② 1:200000 ③ 1:100000	① >5000 ② >20000 ③ >20000
Procymidone	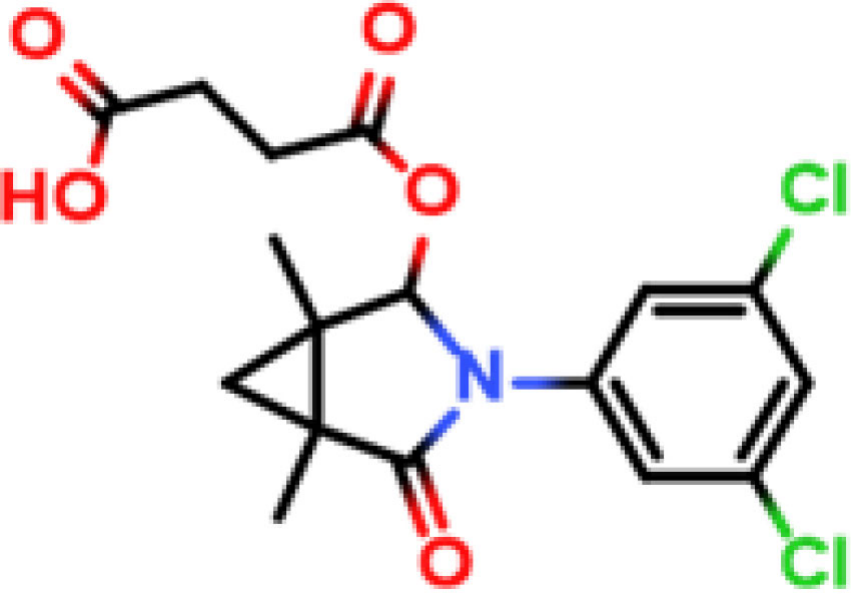	Hapten-cOVA	Hapten-cBSA	Mouse	① 1:250000 ② 1:150000 ③ 1:120000	① >10000 ② >10000 ③ >1000
Chlorantraniliprole	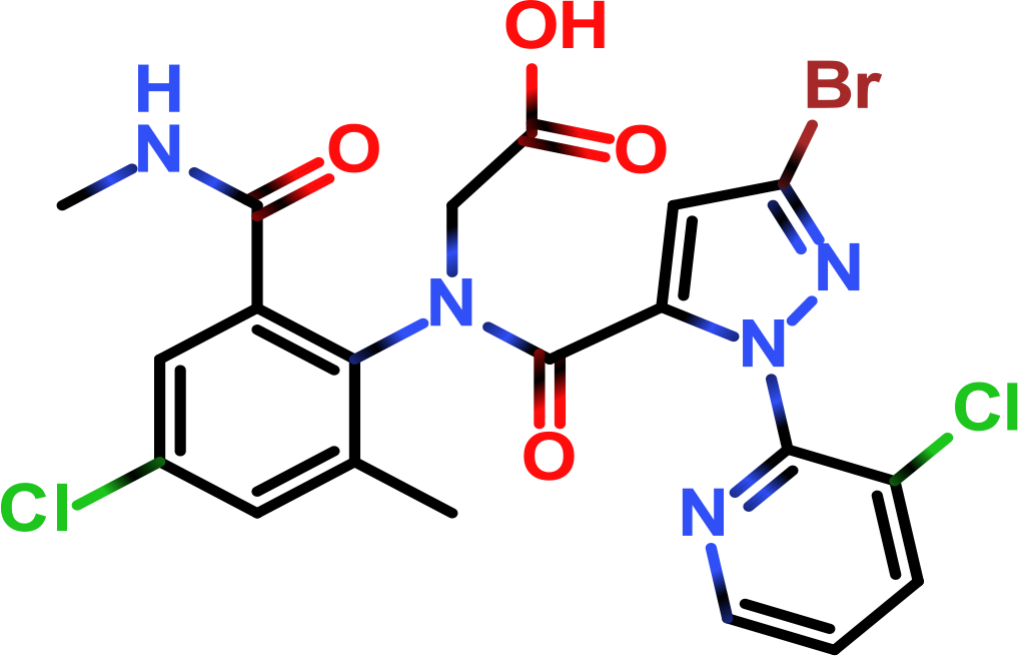	Hapten-cOVA	Hapten-cBSA	Mouse	① 1:150000 ② 1:100000 ③ 1:100000	① >40000 ② >40000 ③ >40000
Bifenthrin	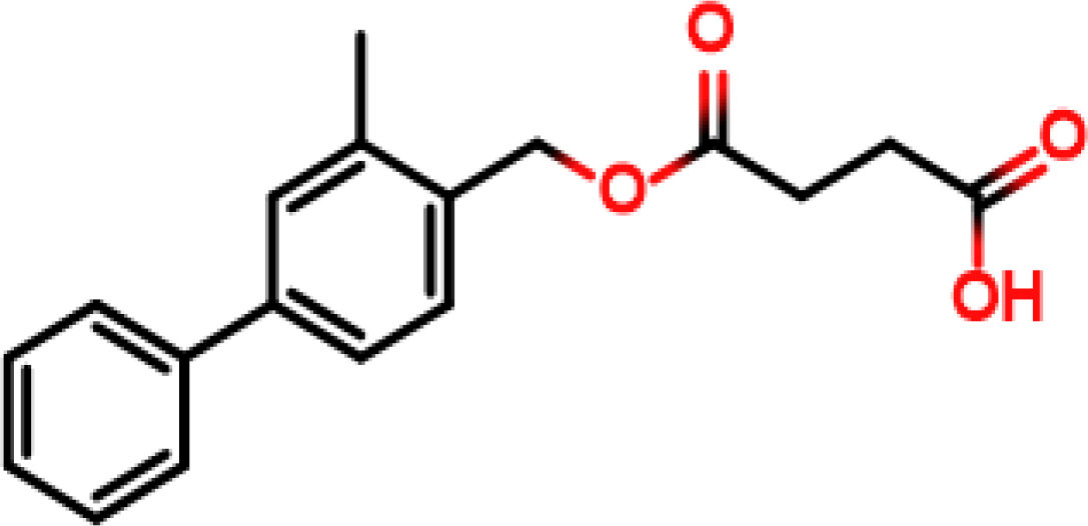	Hapten-cOVA	Hapten-cBSA	Mouse	① 1:300000 ② 1:200000	① 5110 ② 8190
		Hapten-cBSA	Hapten-cOVA	Mouse	① 1:250000	① 8880
Enrofloxacin (ENR)	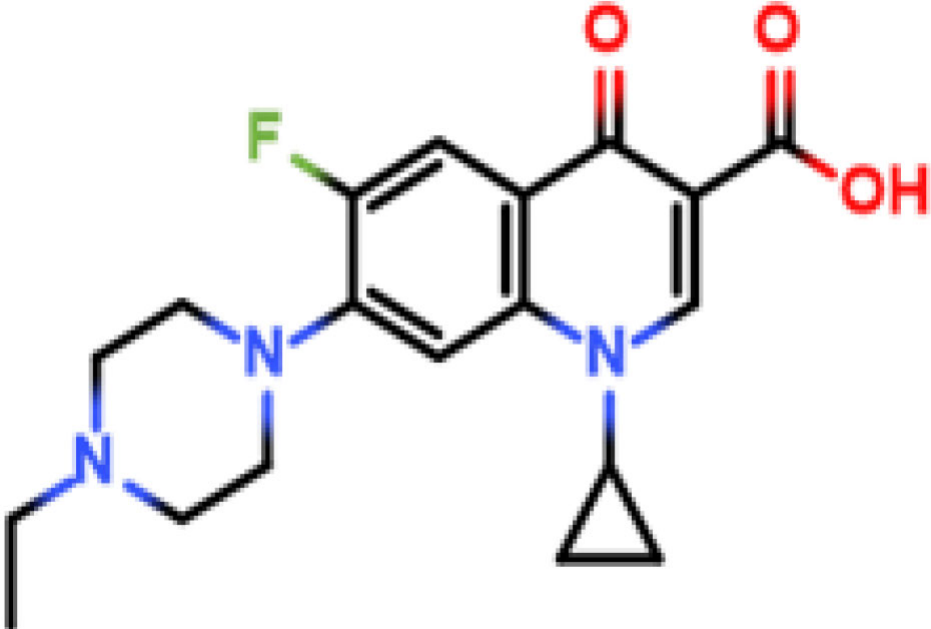	ENR-OVA	ENR-BSA	Mouse	① 1:400000 ② 1:200000	① 500 ② > 1000

a Titer was defined as the reciprocal dilution that results in a 2.1-fold absorbance value for negative serum (14).

b IC_50_ values were the competitor concentrations at which h-alf decreased the absorbance value compared to the absorbance value of no competitor (14).

## Results and discussion

### Properties of cationized complete antigens and the effect on antiserum binding

**Figure 1:**
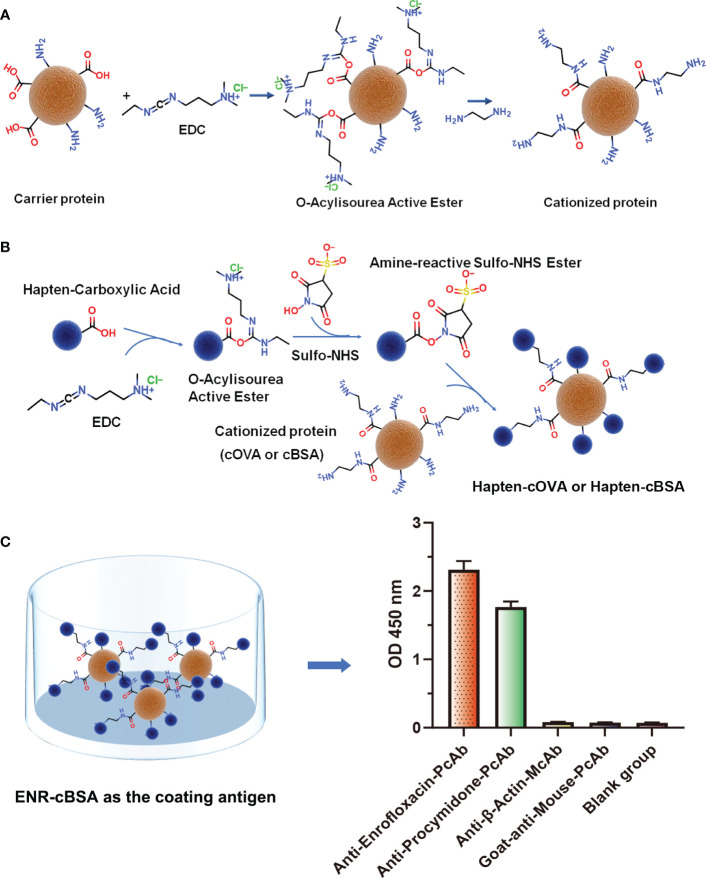
**(A)** The process of preparation of cationized carriers (cOVA or cBSA). **(B)** The process of preparation of complete antigens (hapten-cOVA or hapten-cBSA). **(C)** The binding abilities of four different antisera and antibodies to ENR-cBSA.

Following the typical strategy, BSA and OVA were pretreated by EDA through the EDC-induced reactions before coupling haptens to carrier proteins (34–36) (**Figure 1A**). Converting carboxyl groups to cationized groups (
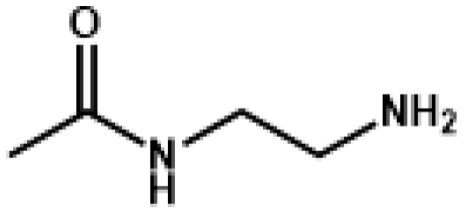
) was considered helpful in preventing crosslinking of carrier proteins and improving the hapten coupling efficiency. The preparation of complete antigens was done using cOVA or cBSA as the carrier by the carbodiimide method (EDC/NHS) (**Figure 1B**). Based on MALDI-TOF/TOF analysis (**Supplementary Figure S1**), the numbers of free amino groups in cationized proteins (cBSA and cOVA) were increased by two to three times, while the number of cationized groups increased by 60 and 43, respectively. The coupling ratio of ENR to cOVA was increased to 10.2/1, which was almost twice that of OVA (5.2/1). The coupling ratio of the ENR to cBSA was increased to 14.7/1, which was almost twice that of BSA (8.2/1) (**Table 2**) and correlated well with previous studies (37, 38). However, the sensitivity of ENR detection by ELISA was not improved with the significantly increased amino groups and hapten–carrier coupling ratios. While the titers of antisera were considerably high, the observed IC_50_ values were inferior (**Table 1**), which demonstrated that the high bindings may not come from the specific antibody to target small molecules. The source and formation of this undesired binding must be explained in detail, and the mechanism should be elucidated to explore the reasons for the inefficiency of antibody preparation.

**Table 2:** The coupling ratios and the number of free amino groups calculated by MALDI-TOF/MS/MS.

Protein	Coupling substance	Coupling Ratios	Number of free amino groups
BSA OVA	——	— —	60 20
cBSA cOVA	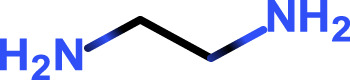	~60 ~43	~120 ~63
ENR-OVA ENR-cOVA ENR-BSA ENR-cBSA	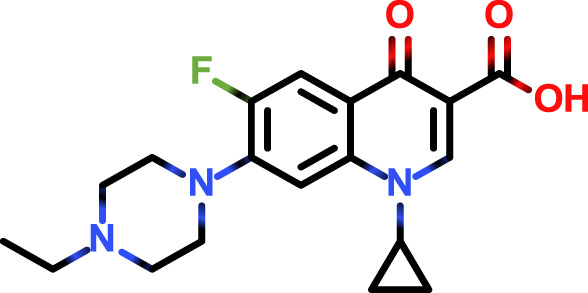	~5.2 ~10.2 ~8.2 ~14.7	~14.8 ~52.8 ~51.8 ~105.3

To further explore the possible source of this undesired binding between antisera and coating antigen, the same concentration of four different antisera and antibodies, including anti-enrofloxacin serum, anti-procymidone serum, anti-β-actin monoclonal antibody, and goat anti-mouse IgG polyclonal antibody were incubated with ENR-cBSA (coating antigen) to explore whether the binding was due to cationized carrier or a cationized process. As shown in **Figure 1C**, something interesting was found: anti-enrofloxacin polyclonal antibody and anti-procymidone polyclonal antibody all have a strong binding with ENR-cBSA. The anti-β-actin monoclonal antibody and goat anti-mouse IgG polyclonal antibody showed no binding activity. The antibodies prepared are supposed to bind only to the corresponding antigen. The anti-procymidone serum should not bind to the ENR coating antigen. Considering both anti-enrofloxacin serum and anti-procymidone serum were generated with cOVA as the carrier protein of immunogen, but anti-β-actin monoclonal antibody and goat anti-mouse IgG polyclonal antibody were generated directly using the macromolecule as immunogens without any modification, the antiserum bound to ENR-cBSA should not be attributed to some nonspecific interactions between coating antigens and IgG, such as the possible electrostatic force induced by a large number of amino groups. Instead, many antibodies seemed to be generated against some unknown epitopes on the coating antigens, and the suspected epitopes corresponding to these antibodies should have been newly formed during protein modification or/and hapten–protein conjugation.

### Exploration of epitopes affecting the efficiency of hapten-specific antibody production

**Figure 2:**
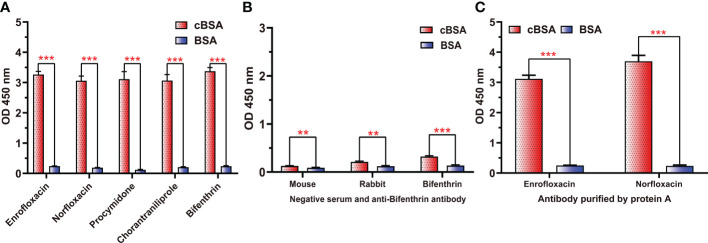
The binding abilities of different antisera and antibodies to cBSA or BSA. **(A)** Five antisera prepared with cOVA as an immune carrier. **(B)** Negative sera and antisera against bifenthrin prepared using OVA as the immune carrier. **(C)** Polyclonal antibodies against ENR and NOR after purification by protein **(A)** ^***^ *p* < 0.001; ^**^ *p* < 0.01—levels of significant difference.

To further demonstrate whether neoepitopes were generated, different antisera prepared from cationized antigens diluted to the same concentrations were used to bind cBSA, and BSA was used as a control. All antisera generated using cOVA as immunogen carrier (anti-enrofloxacin serum, anti-norfloxacin serum, anti-procymidone serum, anti-chlorantraniliprole serum, and anti-bifenthrin serum) exhibited strong binding to cBSA but weak binding to BSA (**Figure 2A**), even after purification by a protein A affinity column to obtain antibodies (**Figure 2C**). Alternatively, all negative antisera and antiserum generated using OVA as the carrier (anti-bifenthrin serum) showed very weak recognition for both cBSA and BSA (**Figure 2B**). These results confirmed the formation of neoepitopes during the process of OVA modification. The cationized carriers mainly use ethylenediamine modification to convert the carboxyl group to the cationized group (
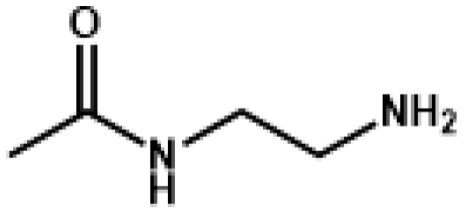
), forming an amide group. The large quantities of amide groups in the immunogen have the potential to generate antibodies as new antigenic determinants. The coating antigens prepared in the same way also contain a large number of amide groups, and the antibodies against amide groups could bind with the coating antigen to show high absorption values.

**Figure 3:**
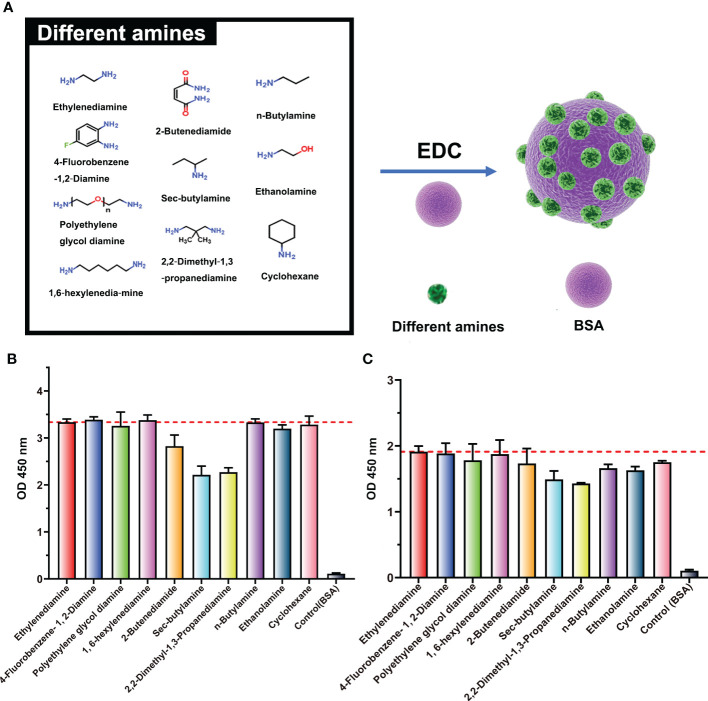
Binding ability of ENR-cOVA antibodies and BSA modified with various amines. **(A)** Structures of BSA modified with different amines by EDC reaction. **(B)** Antibody diluted 5,000-fold. **(C)** Antibody diluted 20,000-fold.

To further confirm the structure of the neoepitope, BSA was pretreated with nine different amines (*n*-butylamine, cholamine, cyclohexylamine, 2-butenediamide, *sec*-butylamine, 2,2-dimethyl-1,3-propanediamine, 4-fluorobenzene, polyethylene glycol diamine, and 1,6-hexylenediamine) and then used as the coating antigen for ELISA analyses. All antigens were bound to the ENR-cOVA polyclonal antibody (purified by the protein A column) at two different dilutions, with efficiency at 70%~100% of that of EDA-treated antigens (**Figure 3**). The spatial structure of these amines is widely disparate (**Supplementary Figure S2**); thus, this observed strong binding should not be due to the crossreactivity of antibodies recognizing similar structures. On the other hand, no matter which amine was used for pretreatment, many amide-containing neoepitopes (
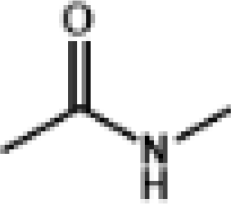
) would have been formed and exposed on protein surfaces. As the only typical structure of all these modified proteins, the amide-containing neoepitopes could reasonably be concluded to be a neoepitope.

### Generation of polyclonal antibody against amide-containing neoepitopes

**Figure 4:**
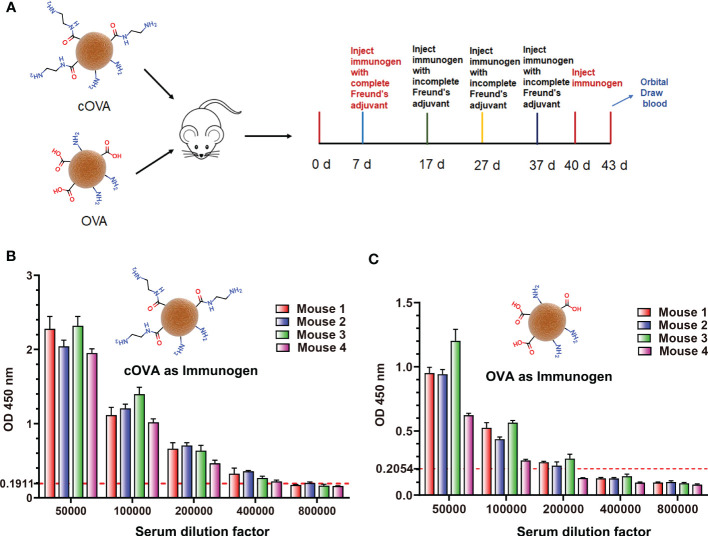
**(A)** The process of preparing antisera against cOVA and OVA. **(B)** Titers of antisera against cOVA; the dotted line is 2.1 times the negative value. **(C)** Titers of antisera against cOVA; the dotted line is 2.1 times the negative value.

**Figure 5:**
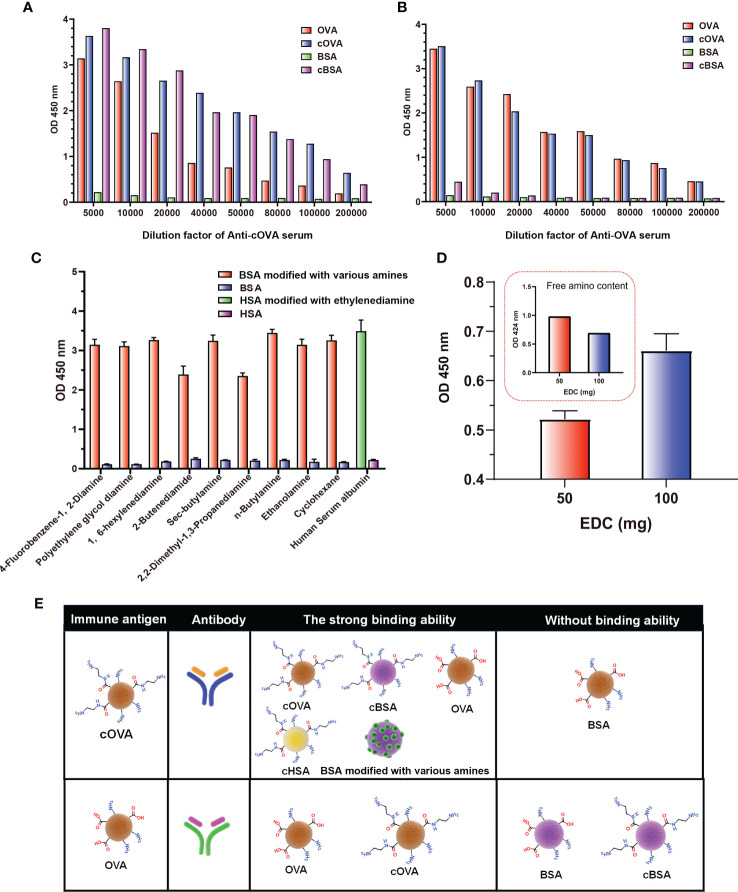
Binding of antisera prepared against cOVA and OVA. **(A)** The binding ability of antisera against cOVA with OVA, cOVA, BSA, and cBSA. **(B)** The binding ability of antisera against OVA with OVA, cOVA, BSA, and cBSA. **(C)** The binding ability of antisera to BSA modified by different amines and HSA modified by EDA. **(D)** The binding ability of antisera against cOVA to self-crosslinking protein and amino amounts of self-crosslinking protein. **(E)** Binding abilities of antibodies against cOVA and OVA to different coating antigens.

According to the above results, the preparation of cationized OVA could form lots of neoepitopes. In order to further verify whether antibodies against neoepitopes could be produced, cOVA was used as an immunogen to immunize the mice, and OVA was the control (**Figure 4A**). After immunization at the same dose, titers of the antisera prepared with cOVA were higher than those with OVA (**Figures 4B, C**). Other research has reported similar results, and the enhanced immunogenicity of cOVA might have resulted from a high isoelectric point (*pI*) (18). Antisera generated against cOVA did not bind to BSA, but they bound to OVA, cOVA, and cBSA, and the bindings were stronger for cBSA and cOVA than OVA (**Figure 5A**). On the other hand, antisera generated against OVA showed similar binding values to OVA and cOVA, with no binding values to BSA or cBSA (**Figure 5B**). Also, they were bound to BSA pretreated with different amines and human serum albumin (HSA) modified with ethylenediamine (**Figure 5C**). At the same time, excessive use of EDC could induce crosslinking of the carrier protein and form some amide-containing neoepitopes due to the higher reactivity between the carboxyl and amino groups (39). The prepared cOVA antiserum showed a high binding ability to the carrier protein treated with a high concentration of EDC; the reduction of the free amino groups provides further evidence of amide bond formation; the free amino acids were measured by trinitrobenzene sulfonic acid (TNBS) method (40) (**Figure 5D**). Thus, it was likely that cOVA had two different types of epitopes: one on native OVA, which induced antibodies binding to both OVA and cOVA, and the other epitope comprising amide-containing neoepitope was formed during the ethylenediamine modification, which induced antibodies recognizing cOVA, cHSA, and BSA modified with different amines (**Figure 5E**).

### Molecular simulations to investigate the effect of amide groups on antigen properties and antibody preparation

**Figure 6:**
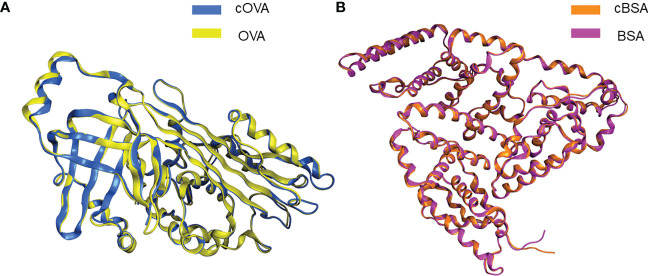
Conformational changes following protein modifications. **(A)** Protein overlay of cOVA and OVA. **(B)** Protein overlay of cBSA and BSA.

**Figure 7:**
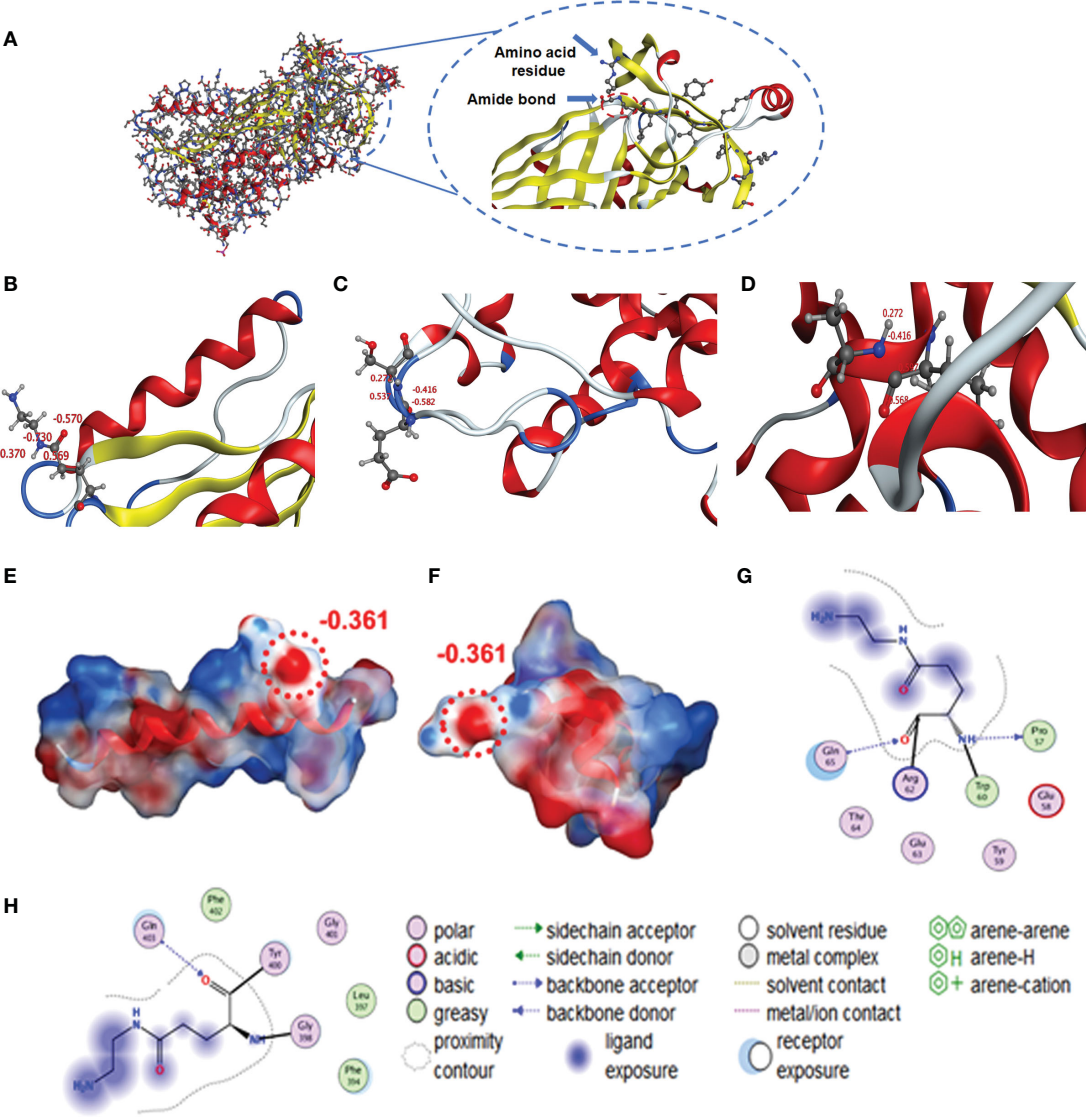
Simulation of amide-containing neoepitopes formed during protein modification. **(A, B)** Amide-containing neoepitopes in the modified amino acid of OVA and charge properties. **(C, D)** Charge properties of amide-containing neoepitopes formed by basic and acidic amino acids in a protein backbone. **(E, F)** Spatial structure and charge properties of amide-containing neoepitopes formed during protein modification (OVA and BSA). **(G, H)** Modified Glu was selected as the ligand to study interactions with amino groups and unique structures.

We used Molecular Operating Environment (MOE 2019.0102) to compare the structural differences between natural and cationized proteins, both for OVA and BSA. No significant changes in protein spatial structure were found after protein stacking (**Figures 6A, B**). The amide group should be a crucial factor in interference to produce hapten-specific antibodies. Almost all amide-containing neoepitopes in the protein backbone were hidden by the tertiary structure of the protein, with amino acid R-groups exposed on the surface (**Figure 7A**). For OVA and BSA, carboxyl groups were mainly provided by aspartic acid (Asp) and glutamic acid (Glu) and induced amide-containing neoepitopes during the modification. When analyzing the Huckel charge distribution, newly synthesized amide-containing neoepitopes during protein modification were demonstrated to possess more electronegativity than the original structures of the protein skeletal structure (**Figure 7B**), which were initially almost electrically neutral or with a small amount of negative charge (**Figures 7C, D**). With the introduction of these amide-containing neoepitopes, the overall electronic properties of the proteins were significantly changed (**Supplementary Figure S3**), and many strong negative charge centers were formed based on the amide-containing neoepitopes on the surface of proteins in complete antigens, which could constitute spatial conformation full enough to act as epitopes (**Figures 7E–H**). Very similar structures were observed in BSA treated with different amines (**Supplementary Figure S4**), which indicated that the newly formed amide-containing neoepitopes were the basis and core of the steric conformation.

**Figure 8:**
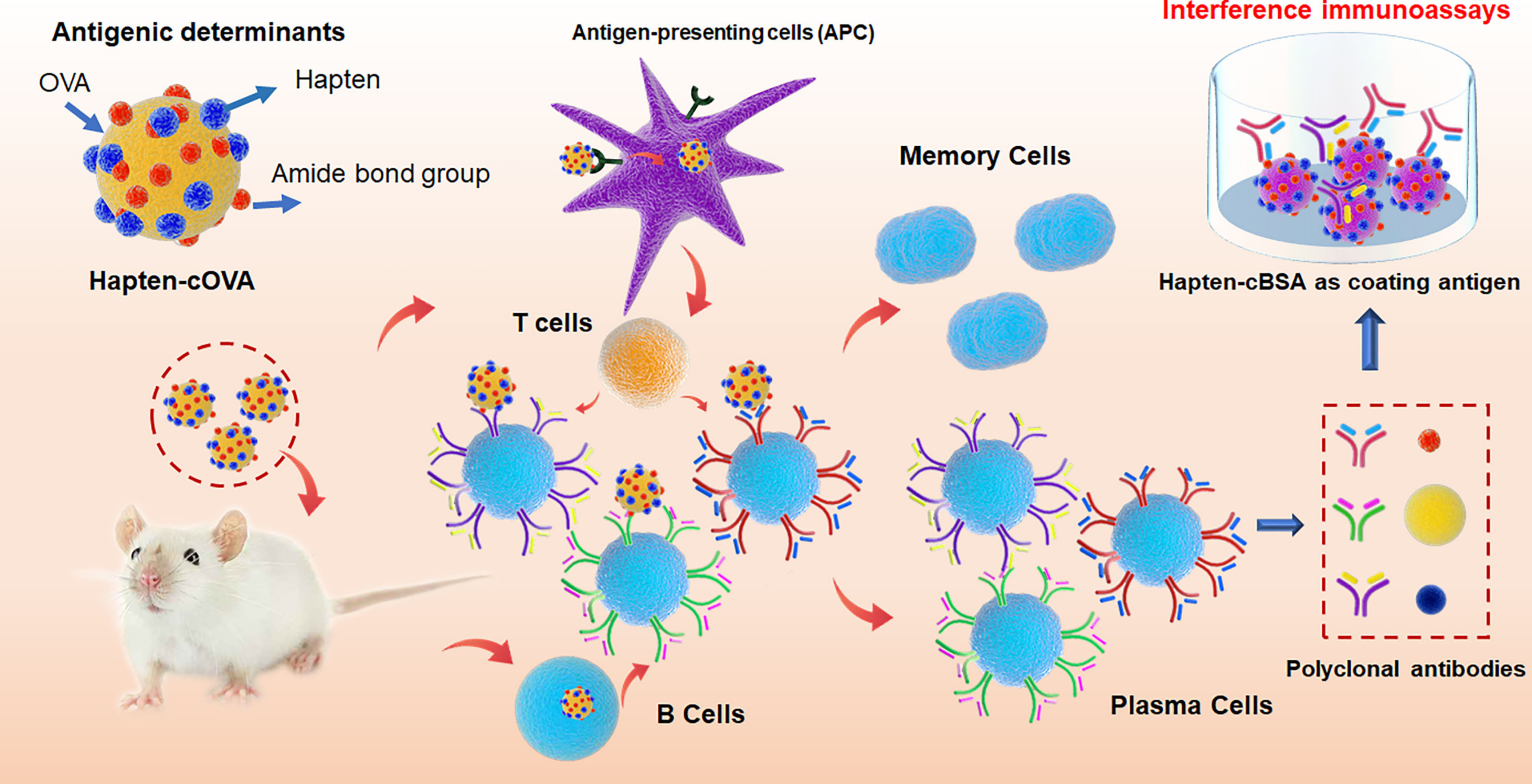
The mechanism model for the effect of amide-containing neoepitopes on the generation of polyclonal antibodies, including antigenic determinants in hapten-cOVA, the production of antibodies against amide-containing neoepitopes, and the mechanism for interference immunoassays.

Some unique properties might significantly enhance the immunogenicity of the amide-containing neoepitopes. The neoepitope could be the key recognition site because of its strong negative charge. Chen et al. found that the amide group in hapten resulted in a significant decrease in specific antibody binding activity, which further supports our conclusion (41). Kavun et al. found that neoepitopes formed *via* peptide–protein conjugation using glutaraldehyde as a covalent crosslinking agent, and the high charge density of Schiff base may be central to the new antigenic epitope. This conclusion was similar to our found amide-containing neoepitopes (42). Therefore, in principle, at least three different antibodies could be generated during the conventional immunization: antibodies for the target hapten, the carrier protein, and the amide-containing neoepitopes. According to calculations of free amino groups and coupling ratios (**Table 2**), the number of these amide-containing neoepitope charge centers was at least four to five times than that of haptens on the complete antigens, and their electronic intensity was evaluated to be ~5-fold than that of hapten-based epitopes. There are approximately 10 amino acid sequences in OVA that can serve as antigenic determinants (43), which is also less than in amide-containing neoepitopes. Such advantages in both immunogenicity and quantity would greatly promote the generation of numerous antibodies that recognize amide-containing neoepitopes and therefore significantly increase the difficulty of screening for antibodies specific to target haptens. Moreover, the phenomenon of antiserum with high titers but low affinity for small molecules is well explained. The specific recognition for target haptens was extensively covered up, which appeared as high titers but poor IC_50_ values. The constructed mechanism model is shown in **Figure 8**. Even under noncationized conditions, it is imaginable that protein self-crosslinking caused by excessive EDC will generate large amounts of amide groups, activating the immune response to produce antibodies and resulting in a decrease in antibody quality.

We have demonstrated through the above analysis that the amide group plays an important role in the overall neoepitope, but the core structure still needs to be explored in further research. Laver et al. found that the epitope of the natural protein had 15–22 amino acids. However, the results of the binding energy analysis showed that five to six residues contributed the highest binding energy, and they were scattered over the surface of the epitope (44). Leder et al. concluded that antiserum to a native protein may crossreact with the corresponding denatured protein. The crossreaction is either a genuine property of the antibodies or caused by antibodies produced against some unfolded protein contaminating the native protein used for immunization (45). However, how many amino acids were contained in the structure of the entire neoepitopes and whether the modification process produces a hidden epitope of the natural protein fold exposed to the surface requires antigen–antibody binding kinetics as well as structural analysis, along with antigen antibody-targeted mutagenesis, and we were unable to reach definitive conclusions in this study. Nevertheless, the antisera immunized by cOVA have a strong binding ability to cOVA, and cBSA may reflect the central role of the acidic amino acid amide group. It is considered that further studies are needed.

Based on this mechanism, affinity purification of the polyclonal antibody with hapten may be a good way to obtain antibodies with high sensitivity. In our previous study, hapten-branched polyethyleneimine was used as an affinity ligand to purify polyclonal antibodies specific to ENR, and the sensitivity of the polyclonal antibody after purification was improved from 19.22 to 1.099 ng/ml (27). However, the best way to solve this problem is to decrease the formation of neoepitopes, such as amide-containing neoepitopes on immunogen.

### Strategy for preparation of antibody with high sensitivity

**Figure 9:**
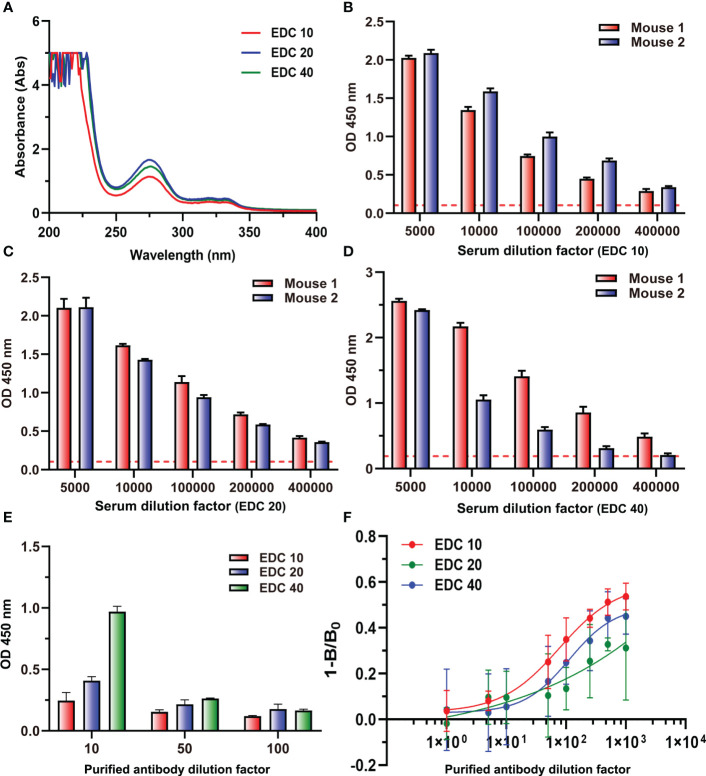
**(A)** The UV–vis spectra of three immunogens. **(B)** The titers of two mouse antisera at EDC 10. **(C)** The titers of two mouse antisera at EDC 20. **(D)** The titers of two mouse antisera at EDC 40. **(E)** The binding values of three purified antibodies to cBSA at three dilution times. **(F)** Competitive inhibition curve of three purified antibodies.

Three immunogens (ENR-OVA) with different gradients of EDC (10, 20, and 40 mg) were prepared and identified by UV spectroscopy at the same concentration. The results in **Figure 9A** showed that the highest coupling ratios were obtained when the content of EDC was 20 mg; the highest content of EDC 40 did not induce the highest coupling ratio but might lead to serious self-crosslinking. When immunized with these three immunogens to prepare antiserum, ELISA results (**Figures 9B–D**) showed that the serum with the highest titers was generated by the immunogen prepared with 20 mg of EDC, which was consistent with coupling ratios. Interestingly, the polyclonal antibody generated by the immunogen prepared with 40 mg of EDC showed a significant binding ability to cBSA even after purification (**Figure 9E**). Also, the ic-ELISA results (**Figure 9F**) showed that the polyclonal antibody generated by the immunogen prepared with the lowest EDC presents the highest sensitivity. These results could be explained by the influence of amide-containing neoepitopes formed during the preparation of immunogen with excessive EDC. On the other hand, it also provided a novel strategy for generating high-quality antibodies by reducing the amount of EDC used in the preparation of immunogens, and the traditional evaluation method of an antibody preparation, which ignores the influences of amide-containing neoepitopes, should be ruled out. It was the first time to elaborate on the problem of antibodies against small molecules with high titers but poor sensitivity and to put forward new strategies for the preparation of antibodies with high sensitivity. The results of this study would provide guidance for the preparation of antibodies for small molecules to be applied in all kinds of fields, such as medical, food, and environment.

According to our studies, several ways could be helpful to produce hapten-specific antibodies and enhance the sensitivity of immunoassays, including: (1) To decrease the formation of amide-containing neoepitopes, it is necessary to optimize the content of coupling regents to a minimum on the premise of ensuring the coupling ratios in the preparation of immunogen and coating antigen. (2) The criterion for a spacer arm design is to form an electroneutral linker to reduce immunogenicity. (3) Two different coupling methods should be used in the preparation of immunogens and coating antigens. Even if numerous antibodies are generated against neoepitopes, the sensitivity of the antibodies specific to target small molecules will not be affected because the coating antigens do not contain such neoepitopes. (4) Adding the structure of neoepitope by linking small molecules with amino acids to neutralize anti-neoepitope antibodies may also be a good approach (42).

## Conclusion

In this study, the reason for the antibodies with high titers but low specificity for small molecules was investigated at both molecular and submolecular levels. It revealed that numerous amide-containing neoepitopes were formed during the modification of carrier proteins and conjugation with haptens induced by EDC or/and NHS. These amide-containing neoepitopes on the surfaces of prepared immunogens have stronger immunogenicity than haptens due to their high negative charges. The production of large numbers of antibodies against neoepitopes significantly interferes with the efficiency of antigen-specific antibody production. To solve this problem, new strategies focusing on adjusting the dose of crosslinking agents were proposed. To our knowledge, it was the first analysis and mechanism of the amide-containing neoepitopes formed during the preparation of immunogen and coating antigen, which obviously interferes with hapten-specific antibody preparation. The results presented in this study are of great scientific significance for the more reasonable and efficient preparation of desirable anti-hapten antibodies in medicine, food, agriculture, and other fields.

## Materials and methods

### Main reagents and instrumentation

Ovalbumin (OVA, Mw ~45,000), *N*-hydroxysuccinimide (NHS), 1-(3-dimethylaminopropyl)-3-ethylcarbodiimide hydrochloride (EDC), complete and incomplete Freund’s adjuvant, human serum albumin (HSA), and sinapinic acid were purchased from Sigma-Aldrich (St. Louis, USA). Bovine serum albumin (BSA, Mw~68,000), protein A beads 4FF prepacked column, anti-β-actin monoclonal antibody, goat anti-mouse IgG polyclonal antibody, goat anti-rabbit IgG/HRP antibody, goat anti-mouse IgG/HRP antibody, TMB single-component substrate solution (3,3′,5,5′-tetramethylbenzidine), and Tween-20 were obtained from Beijing Solarbio Science & Technology Co., Ltd. (Beijing, China). Anhydrous *N*-*N* dimethylformamide (DMF), ethylenediamine, *n*-butylamine, cholamine, cyclohexylamine, 2-butenediamide, *sec*-butylamine, 2,2-dimethyl-1,3-propanediamine, 4-fluorobenzene, polyethylene glycol diamine, and 1,6-hexylenediamine were purchased from Aladdin Reagents Co., Ltd. (Shanghai, China). Polyclonal antibodies against enrofloxacin, norfloxacin, procymidone, chlorantraniliprole, and bifenthrin with cOVA as a carrier protein were prepared by our laboratory. ELISA analyses were performed on 96-well polystyrene “U” bottom microtiter plates (High Binding, Thermo Scientific Inc., Waltham, MA, USA). Skim milk powder was purchased from Becton, Dickinson and Co. (Franklin Lakes, NJ, USA). The 2,5-dihydroxybenzoic acid matrix for MALDI-TOF/MS was obtained from Bruker Daltonics Inc. (Billerica, MA, USA). All other chemicals were of analytical grade and obtained from Sinopharm Chemical Reagent Co. Ltd. (Shanghai, China).

Optical density (OD) was measured using a microplate reader (Thermo Fisher Scientific Co.). The Ultraviolet-Visible (UV–Vis) absorption spectra were obtained using a UV-Vis spectrophotometer (Shimadzu Corp., Kyoto, Japan). A Rapiflex MALDI-TOF/TOF mass spectrometer from Bruker Corp. (Billerica, MA, USA) was used to obtain mass spectrometry results.

### Animals

BalB/c mice (female, 6–8 weeks old) were obtained from Jinan Pengyue Animal Center (Jinan, China). All animal experiments were performed in accordance with the administration of affairs approved by the animal experimental ethics review committee of the College of Food Science and Engineering, Ocean University of China (Number: SPXY2020032017).

### Preparation of cationized carrier proteins and hapten–protein conjugates

The preparation of cationized carrier protein (cOVA and cBSA) was conducted as described by Liu et al. (18) with some modifications. Briefly, 200 µl of EDA, 236 mg of a carrier protein (OVA or BSA), and 256 mg of EDC were dissolved in 10 ml of phosphate-buffered saline (PBS, 0.01 M, pH 7.4) and then incubated at 20°C with shaking at 110 r/min for 12 h. After dialysis in PBS (0.01 M, pH 7.4) for 72 h at 4°C, the products were lyophilized and stored at −20°C. The carrier proteins modified with other amines were prepared in the same process described above.

The conjugation of enrofloxacin (ENR) with different carrier proteins (OVA, cOVA, BSA, and cBSA) was performed by a previously published method (16). Briefly, 20 mg of ENR, 20 mg of NHS, and 20 mg of EDC were dissolved in 1 ml of DMF and incubated at 20°C with shaking at 110 r/min for 12 h. The solution was then added drop by drop into 10 ml of PBS containing 50 mg of carrier protein and then incubated at 4°C for 6–8 h. The products were finally dialyzed in PBS (0.01 M, pH 7.4) at 4°C for 72 h and stored at −20°C for further use. The prepared ENR–protein conjugates were identified by UV–Vis absorption and MALDI-TOF/TOF. The other hapten–protein conjugates were prepared as described above with minor modifications according to the characteristics of the hapten.

### Antisera generation

Antisera against different antigens were generated by the immunization of BalB/C mice. Antigens were dissolved in PBS (2 mg/ml) and emulsified with an equal volume of Freund’s complete adjuvant. After three booster immunizations were given at 10-day intervals with the same dosage, 200 µL of the emulsion was then injected intraperitoneally into BALB/c mouse with the adjuvant changed to incomplete adjuvant. The mouse was finally bled, and the antisera were collected for further use.

### ELISA procedure

The binding ability of prepared polyclonal antibodies was determined by conventional indirect ELISA. The 96-well plate was coated with 100 µl of coating antigen (30 µg/ml dissolved in carbonate buffer solution (CBS)) after incubation at 37°C for 2 h and then blocked with 300 µl of 5% skim milk dissolved in PBS containing 0.05% Tween-20 (PBST) at 37°C for another 2 h. After three times washing with PBST, 100 µl of antisera in appropriate dilution with PBS was added and incubated at 37°C for 1.5 h, followed by three times washing with PBST. Afterward, 100 µl of goat anti-mouse IgG/HRP antibody (1/5,000) in PBS was added and incubated at 37°C for 1 h. The plate was finally washed three times with PBST, and 100 µl of TMB single-component substrate solution was added to each well. After incubation at 37°C for 5 min, the reaction was terminated by adding 50 µl of 2 M H_2_SO_4_. The OD_450_ was measured using a microplate reader to assess the binding ability of polyclonal antibodies.

### Molecular simulation

The molecular simulation and overlay of different carrier proteins were performed in the Molecular Operating Environment (MOE 2019.0102) software on Windows System. The small molecular residues were modified at the acidic amino acid (Glu) site, and then the energy was minimized to obtain the optimal conformation. The modified structure was shown as the ligand in simulations of the molecular surface to show the electrostatics and charges.

### Statistical analysis

Data analysis was performed using GraphPad Prism 8 software. The significant difference analysis was performed by SPSS Statistics v.18.0.

## Data availability statement

The raw data supporting the conclusions of this article will be made available by the authors, without undue reservation.

## Ethics statement

The animal study was reviewed and approved by Animal experimental ethics review committee of College of Food Science and Engineering, Ocean University of China (Number: SPXY2020032017).

## Author contributions

XH, HL, LC, and JS conceived and supervised the project. XH performed the main experiment, developed the molecular simulation, and analyzed data. LW, ZZ, and XW designed the hapten and prepared antibodies against procymidone, chlorantraniliprole, and bifenthrin. XH prepared antibodies against enrofloxacin and norfloxacin. XC and HX helped to perform the MALDI-TOF/TOF experiments. XC and XS conducted chemistry theory experiments. JS and XH wrote the paper with inputs and comments from all co-authors. TP checked the English. All authors have approved the final version of the manuscript.
