# Supplementary material for: Amide-containing neoepitopes: the key factor in the preparation of hapten-specific antibodies and a strategy to overcome
Source: Front Immunol. 2023 Jun 5;14:1144020. doi: 10.3389/fimmu.2023.1144020 (PMC10277511; doi:10.3389/fimmu.2023.1144020)
Supplement: Supplementary file 1 [file DataSheet_1.docx]

**Supporting Information**

**Amide-containing neoepitopes: the key factor in the preparation of hapten-specific antibodies and strategy to overcome**

Xiangning Han^1^, Hong Lin^1^, Xiangfeng Chen^2^, Luefeng Wang^1^, Ziang Zhang^1^, Xiaojing Wei^1^, Xun Sun^1^, Hanyi Xie^2^, Tushar Ramesh Pavase^1^, Limin Cao^1^, Jianxin Sui^1*^

^1^College of Food Science and Engineering, Ocean University of China, 5 Yushan Rd,

Qingdao, China.

^2^Shandong Analysis and Test Center, Qilu University of Technology (Shandong Academy of Sciences), 19 Keyuan Rd, Jinan, China.

*Corresponding authors: Jianxin Sui, E-mail: suijianxin@ouc.edu.cn. Tel: +86-532-82032389.

**Supplementary Text**

**Detection of coupling ratios by MALDI-TOF/TOF**

The coupling ratios of the conjugates were calculated by the following formula.

$N=\frac{Ma-Mb}{Mc}$ …………………………… (1)

N means the coupling ratio, Ma, Mb and Mc mean the mass of the conjugates, the cationized or native proteins and the hapten, respectively. Such as ENR-OVA, the calculated coupling ratios were calculated by N=(46432.159-44565.571)/359.4=5.2.


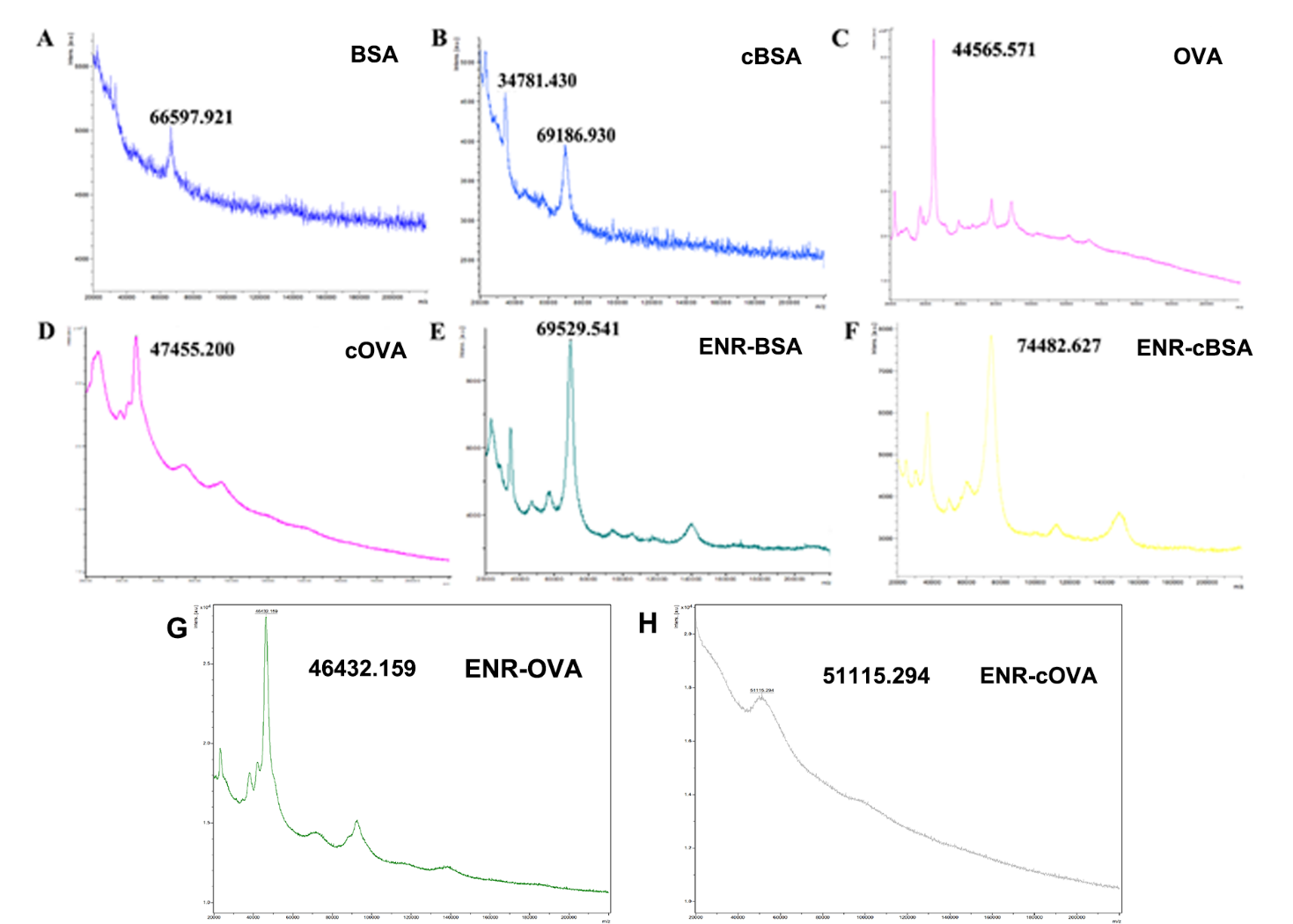


**Figure. S1.** The MOLDI-TOF/TOF results of different proteins. (A) BSA (B) cBSA (C) OVA (D) cOVA (E) ENR-BSA (F) ENR-cBSA (G) ENR-OVA (H) ENR-cOVA

**The Spatial structure of different amines**

**
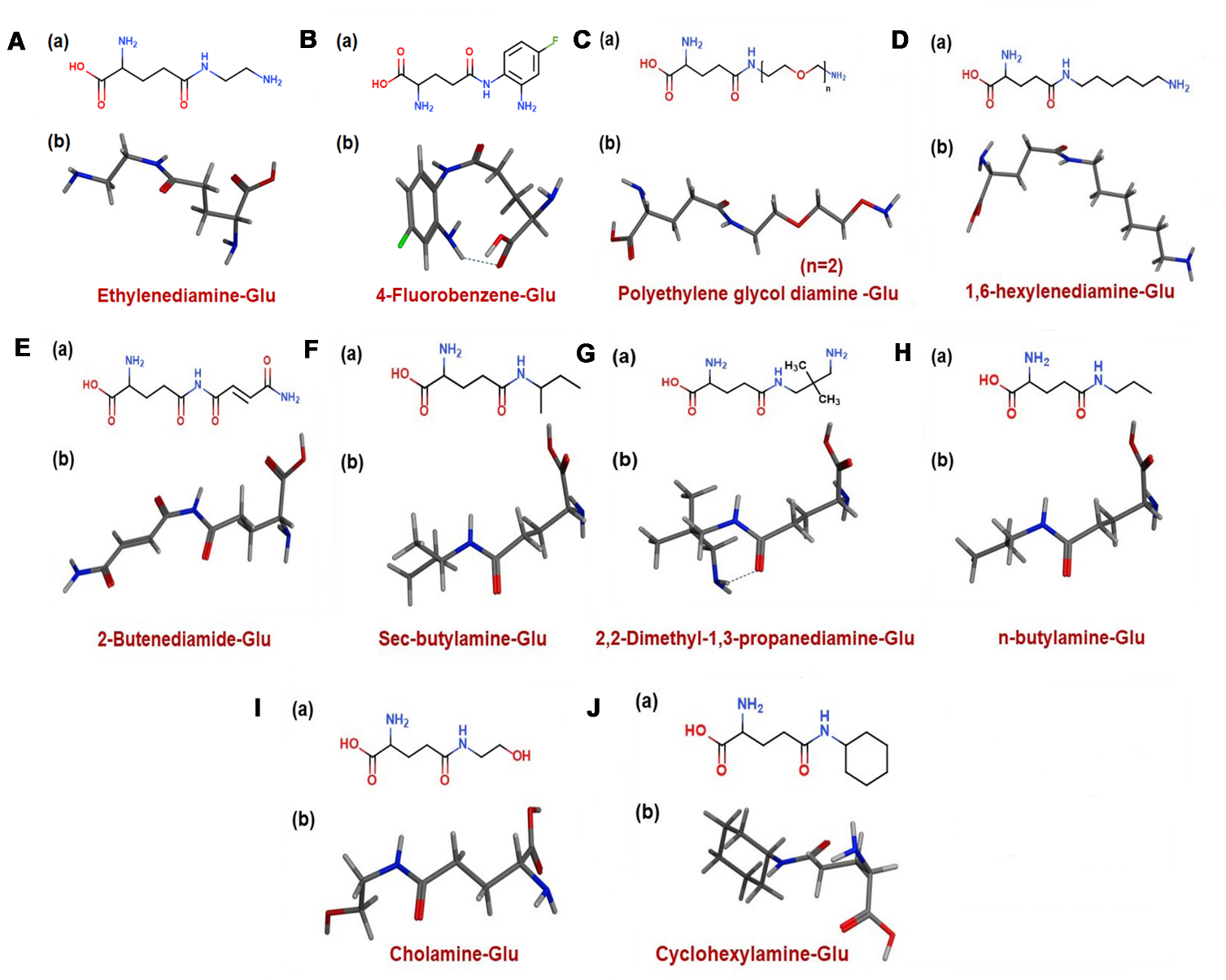
**

**Figure. S2.** The spatial structures of different amines conjugated with glutamate (GLU). (A) Ethylenediamine, (B) 4-fluorobenzene, (C) polyethylene glycol diamine, (D) 1,6-hexylenediamine, (E) 2-butenediamide, (F) sec-butylamine, (G) 2,2-dimethyl-1,3-propanediamine, (H) n-butylamine, (I) cholamine and (J) cyclohexylamine.

**The overall electronic properties of the proteins before and after modification.**


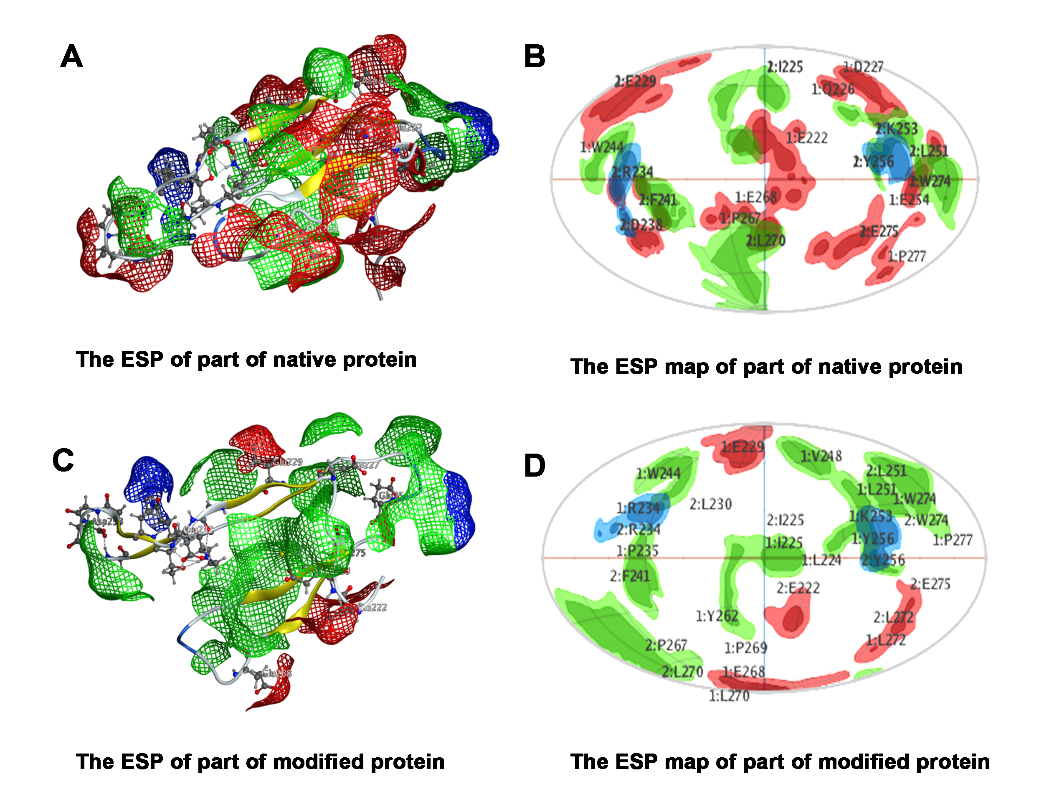


**Figure. S3.** (A) and (B) The negative center of native BSA. (C) and (D) The negative center of cationized BSA.

**The charge distribution of different amines coupled by glutamic acid sites**


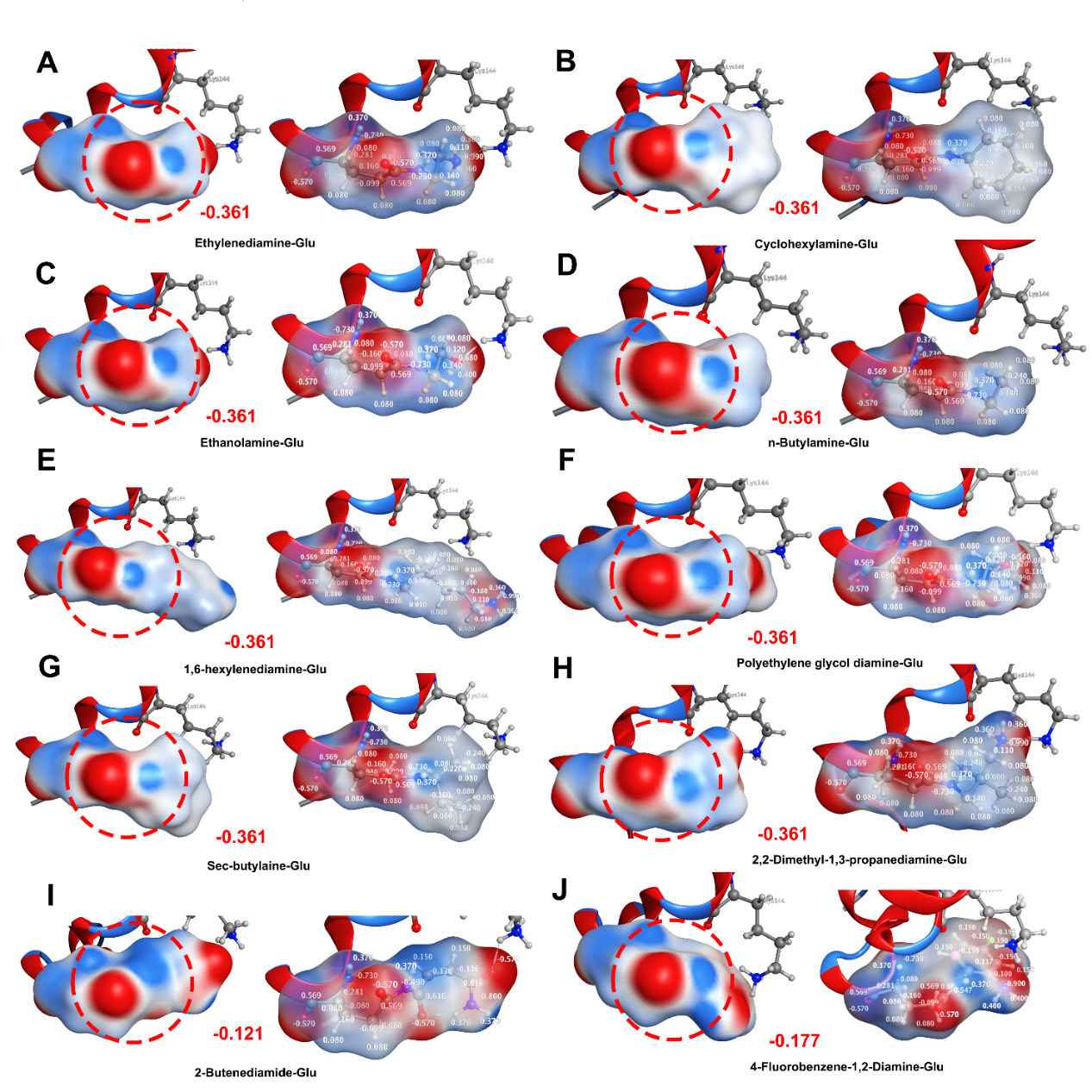
**Figure. S4.** Charge distribution of different amines coupled by glutamic acid sites.
